# Effect of vitamin D on insulin resistance and anthropometric parameters in Type 2 diabetes; a randomized double-blind clinical trial

**DOI:** 10.1186/2008-2231-20-10

**Published:** 2012-08-28

**Authors:** Ramin Heshmat, Ozra Tabatabaei-Malazy, Shabnam Abbaszadeh-Ahranjani, Samimeh Shahbazi, Ghazal Khooshehchin, Fathemeh Bandarian, Bagher Larijani

**Affiliations:** 1 Endocrinology and Metabolism Research Center, Tehran University of Medical Sciences, 5th floor, Dr. Shariati Hospital, North Kargar Ave, 14114 Tehran, Iran

**Keywords:** Diabetes mellitus, Vitamin D, Body mass index, Insulin resistance

## Abstract

**Background & the purpose of the study:**

Prevalence of type 2 diabetes mellitus (T2DM) is increasing worldwide. To reduce its risk and progression, preventive strategies are needed. Vitamin supplementation such as vitamin D is one of the strategies. This study was designed to investigate the effect of injection of vitamin D on insulin resistance and anthropometric parameters in T2DM.

**Methods:**

This randomized double-blind clinical trial was conducted with 42 diabetic patients in two groups; intervention group with single intramuscular injection of 300,000 International Unit (IU) of vitamin D3 and the placebo group. After recording demographic and anthropometric factors (waist circumference, blood pressure and body mass index), fasting blood samples was taken for measurement of blood glucose, 25-hydroxyvitamin D3 (25-OHD3), insulin, glycosylated hemoglobin A1c (HbA1c) and estimation of Homeostasis Model Assessment Index (HOMA) in two times; before study and after three months.

**Results:**

Two groups had similar baseline characteristics (each group = 21 subjects). Three months after vitamin D injection, HbA1c, anthropometric factors and HOMA index in intervention group stayed constant, however, serum 25- OHD3 was significantly increased (p = 0.007).

**Conclusion:**

The present data is not convincing and further studies with large sample sizes are needed to show the definite effect of injection of vitamin D on control of diabetes and its risk.

## Introduction

Prevalence of type 2 diabetes mellitus (T2DM) is increasing worldwide. Nowadays over 360 million people are suffering from diabetes and it is expected that its prevalence reach a staggering 552 million by 2030 [1]. T2DM is defined by impaired glucose tolerance, chronic hyperglycemia, altered insulin secretion, and complications that come from induction of oxidative stress [2]. One of novel strategy toward prevention and control of T2DM is vitamin D supplementation. Besides the role of vitamin D in calcium homeostasis and bone metabolism, other effects also have been proposed for this mineral. Vitamin D is essential for normal insulin secretion in response to glucose and also for maintenance of glucose tolerance [3-6]. In a study performed on 126 healthy adults with normal blood glucose, an association between vitamin D deficiency with beta cell dysfunction and insulin resistance was observed [7]. Such an association has also been established in type 1 diabetes mellitus (T1DM) [8-11]. Though little information is available regarding the association between vitamin D and T2DM [6]. Human and animal studies have shown a negative correlation between serum levels of vitamin D with serum glucose and insulin levels and a positive correlation with insulin sensitivity [5,6]. Several processes are responsible for effect of vitamin D on metabolism of glucose and insulin. Some animal studies have suggested that vitamin D may directly stimulate the insulin secretion from the pancreas. In non-diabetics and also subjects with high blood sugar, consumption of vitamin D supplement improved the insulin secretion [7,12,13]. However, interventional studies have shown conflicting results about the effect of vitamin D supplement on T2DM [6,7,12,14-16]. These differences can be related to difference in race, dosage of vitamin D administration (oral or injection), or duration of usage. To best of our knowledge, no study has assessed the effect of injected mode of vitamin D supplement on diabetes in Iranian population. In this study we evaluated the effect of injected vitamin D supplement on insulin resistance and anthropometric parameters.

## Materials and methods

### Study population

This randomized double-blind clinical trial was performed with 42 diabetic patients, chosen from diabetes clinic of Dr. Shariati Hospital/Tehran. Diabetes was diagnosed according to American Diabetes Association criteria [17]. Inclusion criteria were T2DM subjects’ undertreatment diet and/or oral hypoglycemic agents’ treatment with glycosylated hemoglobin A1c ≤ 7.5%. (HbA1c). Exclusion criteria included: insulin therapy, patient’s reluctance to continue cooperating in every stage of the project, known clinical deficiency of vitamin D, current consumption of vitamin D, multivitamin or calcium, renal failure, nephrotic syndrome, liver failure, known cases of liver cirrhosis or liver dysfunction associated with ascites, hypoalbuminemia, coagulation disorders, and uncontrolled blood pressure. The patient who received the vitamin D injection in the past six months was also excluded from study. After obtaining the written consent, a baseline fasting blood samples was obtain from all the patients. The patients then in a random double blind manner were divided into two groups; intervention group who received a single intramuscular injection of 300000 International Unit (IU) vitamin D3, and placebo group. Each group included 21 subjects and examined in two time points: baseline and three months after vitamin D administration (mean of 92 days) for anthropometric characteristics (systolic and diastolic blood pressure, body mass index (BMI), waist circumference) and biochemical analysis including fasting blood sugar (FBS), 25 - hydroxyvitamin D3 (25-OHD3), insulin, and HbA1c levels. BMI was calculated using following formula: weight (kg)/height^2^ (m). Waist circumference was measured on broadest area between the edge of lower ribs and the iliac crest. Systolic and diastolic blood pressure was measured after 5–10 min rest, on the right arm in sitting position. The study was approved by ethical committee of EMRC and registered in the Iranian Registry of clinical trials (IRCT) with code number of IRCT1138901221414N12.

### Laboratory methods

FBS was measured using photometric assay performed with an autoanalyser Hitachi 902, Pars- Azmon kit/Iran. Insulin and 25-OHD3 levels were measured by ELISA assay using kit Monobound Company (USA) and IDS (USA) respectively. DS5 chromatography Drew Scientific Limited Company (UK) was used for HbA1c measurement. For estimation of insulin resistance the Homeostasis Model Assessment Index (HOMA) based on the following equation was used [18].

HOMA = Fasting plasma insulin (μIU/ml) * fasting blood sugar (mg/dl)/405 The HOMA index value < 3 was considered as normal and ≥ 3 as insulin resistant [19]. 25-OHD3 levels equal to: ≤ 21, 21–29, and ≥ 30 ng/ml were defined as deficient, inadequate, and adequate vitamin D [20,21], respectively. All of the laboratory tests were done in one hormone laboratory.

### Statistical analysis

The normal distribution of data was evaluated by Kolmogrov-Smirnov analytic test. Paired *T*-Test was applied for variables with normal distribution, and Wilcoxon and Mann–Whitney nonparametric tests for others. All the statistical analysis was performed with SPSS software version 15. P ≤ 0.05 was considered as statistically significant.

## Results

Majority of the participants (64%) were composed of female subjects. The age range of patients was 37–79 years with a mean of 56 years and diabetes duration of 5 ± 7 years (mean ± SD). All the patients were under oral hypoglycemic agents (OHA) treatment except in two patients (4.8%) who were on diet therapy for controlling their blood sugar. The OHA treatment of participants was as follow: 16 subjects (38.1%) on combination therapy with metformin and glibenclamide, 9 subjects (21.4%) on glibenclamide, 7 subjects (16.7%) on combination of metformin, glibenclamide, and acarbose, 6 subjects (14.3%) on metformin, and 2 patients (4.8%) on combination of metformin and acarbose. No significantly difference between two groups was present in regards to their anti-diabetic treatment regimen (p = 0.541).

Baseline characteristics of intervention and placebo groups are described in Table 1.

**Table 1:** Baseline anthropometric and biochemical characteristics of patients in both intervention and placebo groups

**Variable**	**Intervention group (n = 21)**	**Placebo group (n = 21)**	**P value**
Age (yr)	56.2 ± 9.3	56.2 ± 11	0.988
Male/Female (%(n))	7 (16.7)/14 (33.3)	8 (19)/13 (31)	1.000
Duration of diabetes (yr)	6.7 ± 4.1	7.9 ± 6.1	0.455
SBP (mmHg)	120.9 ± 9.9	116.6 ± 31.5	0.566
DBP (mmHg)	79.7 ± 10	83.9 ± 18.4	0.331
WC (cm)	92.2 ± 6.7	91.3 ± 8.7	0.945
BMI (kg/m^2^)	27.7 ± 3.4	28.5 ± 3.9	0.484
FBS (mg/dl)	133.5 ± 23.7	138.9 ± 31.3	0.526
HbA1c (%)	6.5 ± 0.9	6.7 ± 0.9	0.598
Insulin (μIU/ml)*	9.2 ± 4.9	10.4 ± 4.7	0.354
HOMA*	3.1 ± 2.3	3.4 ± 1.5	0.145
25-OHD3 (ng/ml) *	46.9 ± 34.7	35.3 ± 36.1	0.212

Legend: SBP: Systolic Blood Pressure, DBP: Diastolic Blood Pressure, WC: Waist Circumference, BMI: Body mass Index, FBS: Fasting Blood Sugar, HbA1c: Glycated Hemoglobin A1c, HOMA: Homeostasis Model Assessment Index, 25-OHD3: 25-Hydroxy vitamin D3.

The results are described as mean ± Standard Deviation (SD).

* The Wilcoxon and Mann–Whitney nonparametric test was used. Paired *T*-Test was used for other variables.

P ≤ 0.05 was considered as statistically significant.

All variables had normal distribution except 25-OHD3 and insulin. Two groups showed no significant differences in anthropometric or clinical characteristics. Table 2 shows the mean differences in both anthropometric and clinical parameters, three months after vitamin D injection between interventional and placebo groups.

**Table 2:** Comparison anthropometric and biochemical characteristics before and three months after study according to mean differences

**Variable**	**Intervention group**	**Placebo group**	**P value**
SBP (mmHg)	1.1 ± 0.2	1.1 ± 0.4	0.763
DBP (mmHg)	0.9 ± 0.2	1 ± 0.2	0.394
WC (cm)	0.3 ± 3.3	0.9 ± 3.8	0.617
BMI (kg/m^2^)	0.08 ± 0.5	−0.3 ± 0.9	0.160
FBS (mg/dl)	16.2 ± 29.2	−9.7 ± 30	0.007**
HbA1c (%)	−0.01 ± 0.9	−0.2 ± 0.9	0.495
Insulin (μIU/ml)*	−0.2 ± 3.6	−2.4 ± 4.2	0.052**
HOMA*	0.2 ± 1.4	−0.9 ± 1.6	0.017**
25-OHD3 (ng/ml) *	22.4 ± 39.9	0.5 ± 17.4	0.009**

Legend: SBP: Systolic Blood Pressure, DBP: Diastolic Blood Pressure, WC: Waist Circumference, BMI: Body mass Index, FBS: Fasting Blood Sugar, HbA1c: Glycated Hemoglobin A1c, HOMA: Homeostasis Model Assessment Index, 25-OHD3: 25-Hydroxy vitamin D3.

The results are described as mean ± Standard Deviation (SD).

* Wilcoxon and Mann–Whitney analytic test was used.

** P ≤ 0.05 was considered statistically significant.

## Discussion

In our study the effect of a single intramuscular injection of 300,000 IU vitamin D (cholecalciferol) in a short period (92 days) in the diabetic patients was evaluated. We found a significant increase in serum 25-OHD3 levels in interventional group without any significant change in level of HbA1c, anthropometric factors, and HOMA index. On the other word, administration of vitamin D in diabetic subjects produced negative effects on control of glycemic status and insulin resistance.

Detecting an increase in serum levels of 25-OHD3 in diabetic subjects following vitamin D injection is in agreement to result of Rahimi et al. [22]. They found a single administration of 600,000 IU cholecalciferol in obese women could significantly increase (p <0.001) the serum 25-OHD3 concentration two weeks after injection. It has been previously reported that a single administration of 100,000 IU of vitamin D is a safe, effective and simple way to increase the serum levels of calcidiol. To ensure the continuity of vitamin D level above the baseline, the injection intervals of not more than two months has been recommended [23]. In our study, the median level of serum 25-OHD3 after 92 days was above the baseline, equal 78 ng/ml.

Series of studies have shown that a positive correlation between low levels of serum 25-OHD3 and impaired insulin sensitivity [24-26], T2DM, hypertension, hyperlipidemia and obesity is exist [7]. An inverse relationship has been reported between serum 25-OHD3 levels and HbA1c, particularly in obese patients [27]. In a cohort study with 17 years follow up, a significant inverse relation between serum levels of serum 25-OHD3 and incidence of T2DM was observed [28]. Indeed a protective role for high levels of plasma vitamin D against T2DM progression has been reported [29]. However, in interventional studies, there are some conflicting results regarding the effect of vitamin D in prevention of T2DM.

Some studies performed that in population with concomitant vitamin D deficiency and impaired glucose tolerance or T2DM, vitamin D supplement was able to correct the insulin secretion and glucose tolerance [12,30], as well as HbA1c [31]. In contrast, some studies failed to show an improvement in levels of blood sugar, glucose tolerance or insulin sensitivity following vitamin D supplement in both diabetic and non-diabetic subjects [4,32-35]. These controversy, or at least part of it, might be due to ethnicity difference in study’s population [6]. The Third National Health and Nutrition Examination Survey (NHANES III) showed an inverse relation between vitamin D status and diabetes in non-Hispanic whites and Mexican Americans, but not in non-Hispanic blacks [36]. Other reasons for discrepancy seen in different studies might be related to duration of study, sample size, and lack of prospective studies or clinical trials specifically aimed to evaluate the effect of vitamin D deficiency on the incidence of T2DM [37]. Dosage or formulation of intake vitamin D may also be considered. Conflicting results on metabolism of glucose and insulin [4,12-14,30,33,34,38-42] has been observed by administration of different forms of vitamin D such as cholecalciferol, orgocalciferol, and active form of this vitamin.

In our study the baseline level of HOMA index in two groups were similar. Interestingly, no remarkable changes in HOMA index following vitamin D injection in intervention group was found, while in placebo group a significant decrease at the end of study was noticed (mean difference = −0.09). In contrast to our result, in a study performed with 33 non-diabetics vitamin D deficient patients, two oral doses of 100,000 IU cholecalciferol corrected the vitamin D deficiency after two weeks without affecting on the glucose or insulin levels [43]. In the other study with 32 diabetic patients under metformin and bedtime insulin treatment, consumption of 40,000 IU weekly cholecalciferol for 6 months had no effect on fasting blood sugar, insulin, c-peptide and HbA1c compared with placebo group [44]. One assumption could be that since insulin secretion is a calcium dependent process, it can indirectly be influenced by effect of vitamin D on calcium absorption. Although a single injection of vitamin D caused significant changes in serum levels of vitamin D, the insulin resistant status has become worsen [44]. On the other word, for improvement of insulin resistant we need to consume a combination of calcium and vitamin D. Meta-analysis of observational and interventional studies have shown that insufficient supplement of vitamin D and calcium has a negative effect on blood glucose while combination therapy was more useful on obtaining optimal glucose metabolism [37]. The observed changes in blood sugar and insulin level between our two groups three months after intervention might be related to difference in their nutritional status. We cannot exactly address this assumption because of unfilled Food Frequency Questionnaire (FFQ) for patients in this study and also because of unawareness of amount of calcium intake in diet of our subjects. It seems that the effect of vitamin D on glucose intolerance is more prominent in vitamin D deficient patients [45]. Due to our small sample size in each group, it is impossible to classify our patients according to their vitamin D status. Similar to our study, one study evaluated three diabetic vitamin D deficient patients after receiving a single intramuscular injection of 300,000 IU orgocalciferol. In this study, three months after injection, undesirable effects on blood sugar and insulin resistance was observed [15].

Obesity is determined as one of the proven risk factors for T2DM. There are several reports that shown an inverse relationship between vitamin D levels and obesity, even in different definition of obesity according to weight, BMI and waist circumference [46-50]. This correlation has been seen in both sexes who were not sever obese [51] and is not depend on dose of vitamin D supplements provided for the patients [52]. However in our study such an association between vitamin D levels and BMI or WC was not found. In New Zealand study [36] also such a correlation was not observed.

One study assessed the results of 14 interventional studies about the effect of vitamin D administration on glucose metabolism and insulin resistance in non-diabetic and diabetic patients [43]. They concluded that the most of these studies could not clearly determine an effective change in blood sugar concentrations following administration of vitamin D. Additionally, by these studies, it is difficult to clear that whether vitamin D or calcium therapy are effective in prevention of diabetes or their effects are presented just in subjects who are at risked to diabetes development. In a systematic review [37] summarizing clinical trials focusing on effect of vitamin D and or calcium in prevention of metabolic syndrome and T2DM, the authors concluded that by these small clinical studies or using post hoc analysis it is difficult to describe whether vitamin D or calcium is effective in prevention of T2DM or its effect is more visible just in people at risk of T2DM. On the other word epidemiologic studies suggested the relationship between low level of vitamin D and impaired glucose tolerance, but intervention studies showed the conflicting results [53].

Most diabetic subjects also suffer from hypertension [54]. Cross-sectional studies have shown an inverse relation between serum levels of vitamin D (even at normal levels) with blood pressure in people with high or normal blood pressure [55,56]. In a large cohort study [57] the relative risk of progression of hypertension in low levels of 25-OHD3 during 4–8 years follow up, was 6.1 in men and 2.7 in women compared with individuals with normal levels of vitamin D. In an interventional study [58] was found the combined consumption of vitamin D and calcium in elderly women with low levels of 25-OHD3, could produce significant reduction in systolic blood pressure (mean 5 mmHg) and in heart rate comparison calcium supplement alone. In our study, administration of vitamin D had no significant effect on systolic or diastolic blood pressure.

Our study had some differences with other studies; all of our patients were diabetic and none of them were undertreatment with insulin. Moreover, the duration of follow up and method of our study was different from previous studies. One assumption for not seeing the effect of vitamin D in our study would be that three months is not enough to see the effects of vitamin D therapy on metabolism of glucose and insulin, though the positive expected effects from vitamin D to increase serum 25-OHD3 levels is produced. It should be noted that affecting the serum levels of vitamin D by supplement consumption can be achieved in times shorter than even a day after single intravenous administration of 1, 25-dihydroxy D [39]. One of limitation in our study was our small sample size. Additionally, our patients were evaluated regardless of their baseline levels of 25-OHD3, calcium and phosphor or unfilled FFQ. Laboratory assays used to determine the circulating level of vitamin D was our another limitation. There are different assays to measure 25-OHD3 levels (59). Immunoassay and Competitive Protein Binding assay are suitable methods; however, LC-MS method that is capable of simultaneously measuring 25-OHD2 and 25-OHD3 concentration is more convenient [58]. Future studies to evaluate effects of concomitant consumption of vitamin D and calcium, effect of vitamin D in patients with vitamin D insufficiency, along with attention to larger sample sizes is suggested.

## Conclusion

Based on previous results; by this study we cannot be sure the effect of single injection of vitamin D on diabetes control and improvement of insulin resistance. Taken our data together available information warrants exploring the possibility that vitamin D in combination with calcium supplementation can be more efficient in reducing T2DM risk than intake of vitamin D alone. Moreover further studies are needed to show the definite effect of injection of vitamin D on control of diabetes and its risk.

## Competing interests

The author(s) declare that they have no conflict of interest.

## Authors’ contributions

Heshmat R coordinated the design and analyzed the data; Tabatabaei-Malazy O conceived the study, collected and analyzed the data, drafted and edited the manuscript, Abbaszadeh-Ahranjani SH drafted the manuscript; Shahbazi cooperated in design and collection of data; Khooshehchin GH coordinated the Lab. data; Bandarian F collected the data; Larijani B conceived the study. All authors read and approved the final manuscript.
